# Food anaphylaxis and augmentation factors – data of the German-speaking anaphylaxis registry

**DOI:** 10.1186/2045-7022-1-S1-P50

**Published:** 2011-08-12

**Authors:** Stephanie Hompes, Franziska Rueff, Scherer Kathrin, Lars Lange, Alice Köhli, Katja Nemat, Nicolaus Schwerk, Margitta Worm

**Affiliations:** 1 Charité - Universitätsmedizin, Department of Dermatology and Allergy, Berlin, Germany; 2 Ludwig-Maximilians-University, Department of Dermatology and Allergology, Munich, Germany; 3 University Hospital, Allergy Unit, Department of Dermatology, Basel, Switzerland; 4 St. Marien-Hospital, Children's Hospital, Bonn, Germany; 5 University Children's Hospital, Department of Allergy, Zurich, Switzerland; 6 Universitätsklinikum Carl Gustav Carus, Department of Paediatrics, Dresden, Germany; 7 Medical School, Department of Paediatrics, Hannover, Germany

## Background

Anaphylaxis is a severe, life-threatening systemic hypersensitivity reaction. The anaphylaxis-registry collects data of patients with severe allergic reactions from 86 allergy centres in Germany, Austria and Switzerland. The present analysis was performed to get more insight into the elicitors and circumstances of food-induced anaphylaxis.

## Methods

The data are delivered by a password-controlled internet-based-questionnaire. Only severe reactions with pulmonary and/or cardiovascular symptoms are accepted. After plausibility approach 2115 anaphylactic reactions, registered from July 2006 until October 2010, were included in the data analysis set.

## Results

Of the 2115 anaphylactic reactions 494 cases (23%) were caused by food. These were 232 reactions in children and adolescents (range: 2 months – 17 years, median: 4 years; 65% male) and 262 reactions in adults (range: 18 – 82 years, median: 39 years; 31% male).The most common triggers among children were peanuts (n = 50), cow's milk (n = 27), hazelnuts (n = 19) and henÂ´s egg (n = 14). Among adults the following triggers were registered most frequently: wheat flour (n = 29, in 25 cases in combination with exercise), soybeans (n = 19), celery (n = 16), shellfish (n = 15) and hazelnuts (n =15).

At least one augmentation factor was observed in 29% of all patients with food anaphylaxis. Drugs (mainly NSAID and beta-blocker) were suspected in 14%, exercise in 10%, alcohol and psychological stress in 4% each of the food cases. As further possible factors acute infection, food-additives and menstruation were reported in less than 2% of the cases. Drugs were most commonly combined with vegetables (celery, carrot) and exercise with wheat flour followed by tree nuts.

## Conclusions

The elicitor profile of food dependent anaphylaxis is age dependent. Possible augmentation factors like drugs and physical exercise were observed in up to one third of anaphylactic patients. Their underlying mechanism should be explored in more detail in future studies.

